# Man with a swollen mass on sacrococcygeal region

**DOI:** 10.11604/pamj.2018.29.10.14447

**Published:** 2018-01-04

**Authors:** Fred Bernardes Filho, Andreia de Oliveira Alves

**Affiliations:** 1 Dermatology Division, Department of Medical Clinics, Ribeirão Preto Medical School, University of São Paulo, Ribeirão Preto, Brazil; 2 Medical School, Centro Universitário Barão de Mauá, Ribeirão Preto, São Paulo, Brazil

**Keywords:** Pilonidal sinus, furunculosis, rectal fistula

## Image in medicine

A 43-year-old male patient presented to the emergency department with 8 days of a painful swollen mass on sacrococcygeal region. He had no personal or family medical conditions and was receiving cephalexin 500 mg four times a day two days ago. Examination revealed an erythematous, fluctuant, swollen mass on sacrococcygeal region and an orifice at the buttock cleft (A). He was afebrile and nontoxic. Laboratory study results were unremarkable. Incision, drainage, currettage were made, leaving the surgical wound open for secondary healing. The diagnosis of pilonidal cyst with abscess was performed. Pilonidal sinus probably derives from the perineal pilosebaceous unit and precursor pits associated with trapped hairs. The penetrating hairs may cause a foreign-body giant cell reaction, sometimes with secondary bacterial infection, with can cause a sudden onset of pilonidal abscess. It occurs more commonly in individuals with stiff dark or auburn hair. There are four clinical presentation of the disease: symptomatic, acute pilonidal abscess, chronic fistulizing form or complex pilonidal sinus disease. Chronic fistulizant form is the most common clinical presentation. The sacrococcygeal location is the most common site but it can occur on the púbis, anterior perineum and very rarely, the penis. Asymptomatic pits do not require treatment. For acute abscesses, the options available are: aspiration followed by treatment with antibiotics and later curative intent surgery; drainage without curettage; drainage and curettage.

**Figure 1:**
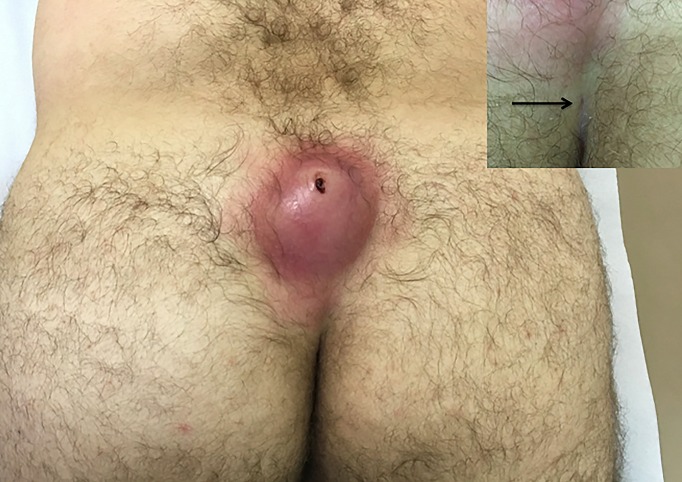
erythematous, fluctuant, swollen mass on sacrococcygeal region and an orifice (arrow) at the buttock cleft in close

