# Corrigendum: Man with a swollen mass on sacrococcygeal region

**DOI:** 10.11604/pamj.2018.30.60.16069

**Published:** 2018-05-28

**Authors:** Fred Bernardes Filho

**Affiliations:** 1 Dermatology Division, Department of Medical Clinics, Ribeirão Preto Medical School, University of São Paulo, Ribeirão Preto, Brazil

**Keywords:** Corrigendum, pilonidal sinus, furunculosis, rectal fistula

## Abstract

This corrigendum corrects article “Man with a swollen mass on sacrococcygeal region” and its publication references ie The Pan African Medical Journal. 2018;29:10. doi:10.11604/pamj.2018.29.10.14447.

## Corrigendum

The original version of this article [1] had the author’s name wrongly abbreviated on pubmed ie Filho FB instead of Bernardes Filho F.
